# Efficient Hydrogen Delivery for Microbial Electrosynthesis via 3D-Printed Cathodes

**DOI:** 10.3389/fmicb.2021.696473

**Published:** 2021-08-03

**Authors:** Frauke Kracke, Jörg S. Deutzmann, Buddhinie S. Jayathilake, Simon H. Pang, Swetha Chandrasekaran, Sarah E. Baker, Alfred M. Spormann

**Affiliations:** ^1^Department of Civil and Environmental Engineering, Stanford University, Stanford, CA, United States; ^2^Materials Science Division, Physical and Life Sciences Directorate, Lawrence Livermore National Laboratory, Livermore, CA, United States; ^3^Department of Chemical Engineering, Stanford University, Stanford, CA, United States

**Keywords:** microbial electrosynthesis, gas fermentation, bioelectrochemical system, hydrogen mass transfer, current density, 3D-printing, additive manufacturing (3D printing)

## Abstract

The efficient delivery of electrochemically *in situ* produced H_2_ can be a key advantage of microbial electrosynthesis over traditional gas fermentation. However, the technical details of how to supply large amounts of electric current per volume in a biocompatible manner remain unresolved. Here, we explored for the first time the flexibility of complex 3D-printed custom electrodes to fine tune H_2_ delivery during microbial electrosynthesis. Using a model system for H_2_-mediated electromethanogenesis comprised of 3D fabricated carbon aerogel cathodes plated with nickel-molybdenum and *Methanococcus maripaludis*, we showed that novel 3D-printed cathodes facilitated sustained and efficient electromethanogenesis from electricity and CO_2_ at an unprecedented volumetric production rate of 2.2 L_*CH4*_ /L_*catholyte*_/day and at a coulombic efficiency of 99%. Importantly, our experiments revealed that the efficiency of this process strongly depends on the current density. At identical total current supplied, larger surface area cathodes enabled higher methane production and minimized escape of H_2_. Specifically, low current density (<1 mA/cm^2^) enabled by high surface area cathodes was found to be critical for fast start-up times of the microbial culture, stable *steady state* performance, and high coulombic efficiencies. Our data demonstrate that 3D-printing of electrodes presents a promising design tool to mitigate effects of bubble formation and local pH gradients within the boundary layer and, thus, resolve key critical limitations for *in situ* electron delivery in microbial electrosynthesis.

## Introduction

Bioelectrochemical technologies represent a key platform for recycling of CO_2_ into useful fuels and chemicals to enable a circular carbon economy (Harnisch et al., 2015; Parag and Sovacool, 2016; De Luna et al., 2019). For bioelectrochemical CO_2_ conversion to operate at industrial scale, two critical performance metrics need to be achieved: (1) high reaction rates, requiring current densities in the range of 100–1,000 mA/cm^2^ and (2) a high selectivity for production of the target compound (>90%) (Bushuyev et al., 2018; De Luna et al., 2019). In this context, microbial electrosynthesis (MES) is particularly promising as biological syntheses are highly selective, including for complex, multi-carbon compounds. Such high selectivity is achieved when using strictly anaerobic microorganisms that typically utilize H_2_, HCO_2_^–^, or CO as catabolic electron source and convert these electrons at a selectivity of >90% to specific catabolic end products (Daniell et al., 2016). So far, the volumetric electron supply in MES has been limited to current densities in the range of 1 mA/cm^2^, with rates up to 20 mA/cm^2^ only reported for projected surface areas that widely underestimate the actual active electrode surface (Prévoteau et al., 2020). Therefore, a key improvement target for transferring this technology from laboratory to larger scale is increasing the current density to maximize the electron feed per volume (Dessì et al., 2020; Jourdin et al., 2020). Hydrogen-driven MES is particularly promising as H_2_ can be electrochemically produced at high rates and 100% selectivity using inexpensive materials and is used by a wide variety of anaerobic microorganisms (Blanchet et al., 2015; Kracke et al., 2019). In traditional microbial gas fermentation, high volumetric production rates are achieved but the handling and dispersion of sparingly soluble H_2_ as electron carrier is a major challenge (Liew et al., 2013). MES is similar to H_2_/CO_2_-gas fermentation, with the important difference that in MES H_2_ is formed *in situ* at the site of microbial consumption and, therefore, circumvents the challenges of introducing bulk H_2_ gas via extensive purging and mixing. Further, the electrochemical production of H_2_ as intermediate provides a platform for electricity storage by converting electrical into chemical energy in form of “green methane” (bio-power-to-gas), which can be used in the existing natural gas infrastructure.

Over the past decade, diverse cathodes have been purposely build or modified to enhance MES processes by improved properties such as increasing hydrogen formation (Zhang et al., 2013; Aryal et al., 2017; Kracke et al., 2019). Cathodes with intrinsically high surface area, such as carbon granules, cloth, fiber, or reticulated vitreous carbon, have emerged as particularly useful to improve the process performance by retention of high bacteria loadings on electrodes and improved start up times (Zhang et al., 2013; Aryal et al., 2017; Kracke et al., 2019). However, a systematic variation of surface area and the relationship between surface and current densities has not been explored so far.

Additive manufacturing, commonly known as 3D-printing, has been a major driver of innovation in industrial manufacturing and could prove to be revolutionary for the field of MES by offering flexibility in complex and custom design of electrochemically active materials (Barnatt, 2014; Lee et al., 2019). The technology enables the rapid development and testing of customized electrode and reactor prototypes and has recently been successfully applied for improved electrochemical carbon dioxide reduction (Corral et al., 2021). In addition to the fabrication of reactor components, 3D-printing is particularly interesting for the production of next generation electrodes as the technology enables the design of high surface area materials with possible structuring up to nanometer-scale via post-treatment methods (Lee et al., 2019). Complex custom designs can be realized, such as incorporation of flow channels to make high surface area per volume accessible, while high conductivity over large electrodes is provided via incorporation of a metallic core. One option to manufacture conductive metal substrates is the printing of metal powder (Kassym and Perveen, 2020). In addition, the direct electrochemical 3D-printing of metallic materials through electrochemical reduction of metal ions from solutions onto 3D-printed conductive substrates is in development (Chen et al., 2017). First studies in the field of bioelectrochemical technologies have explored the 3D printing of electrodes for microbial fuel cell applications (Chung and Dhar, 2021). This includes the printing of metallic, porous 3D-structured materials (Calignano et al., 2015; Zhou et al., 2017), conductive polymeric materials with subsequent surface modifications or carbonisation to increase conductivity and bio-compatibility (You et al., 2017; Bian et al., 2018; Pumera, 2019), as well as the 3D printing of active bio-electrodes by direct incorporation of living bacteria (*Shewanella oneidensis* MR-1) into a printable ink (Freyman et al., 2020). Importantly, in a recent study He et al. report the development of a 3D-printed graphene oxide aerogel anode, which achieved record volumetric current output using a pure culture of *Geobacter sulfurreducens* (He et al., 2021). The authors attribute the performance increase to improved mass transfer properties of the material (compared to carbon felt) enabled by hierarchical pores in their 3D-printed anode (He et al., 2021). These results are a promising indication that 3D-printing presents a suitable tool to address the mass transfer limitation in hydrogen driven microbial CO_2_ reduction.

Here, we demonstrate, to our knowledge, the first use of cathodes fabricated via 3D-printing for microbial electrosynthesis. We manufactured 3D-electrodes with varying ratios of surface area to volume and used these to study the effect of *in situ* H_2_ supply as a function of current density on electromethanogenesis using the methanogen *Methanococcus maripaludis* as model system.

## Materials and Methods

### Cathode Fabrication

**FIGURE 1:**
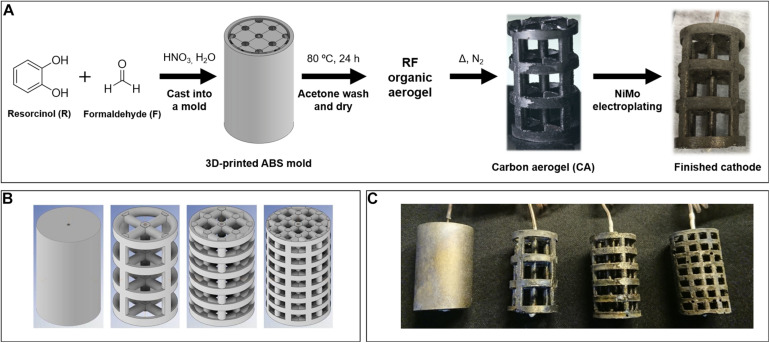
Additive manufacturing process of the NiMo-plated carbon aerogel cathodes **(A)**. Visualization of computational models of inversed ABS molds for 3D-printing **(B)** and photograph of one set of finished NiMo-plated carbon aerogel cathodes of varying surface areas **(C)**. The geometric surface areas of the different cathodes are from left to right 39, 55, 86, and 111 cm^2^.

Carbon aerogel (CA) cathodes coated with a NiMo-alloy were used to enable the fabrication of highly conductive, hydrogen evolving cathodes via additive manufacturing (Chandrasekaran et al., 2018; Kracke et al., 2019). The base CAs were prepared from resorcinol and formaldehyde gels, which were poured into custom 3D-printed acrylonitrile-butadiene-styrene (ABS) molds formed as the inverse of our desired final shape (see Figure 1A). After thermal curing of the gel, the mold was removed by dissolving in acetone, leaving the complex shaped raw organic aerogel, which was carbonized at high temperature in inert atmosphere to yield the CA cathodes. To obtain complex shape casts, acrylonitrile-butadiene-styrene (ABS) molds were printed on a Lulzbot Taz 6 fused filament 3-D printer. Cylindrical lattice electrodes were constructed with 3 mm strut thickness, where the geometric surface area of the electrode was controlled by the density of struts (and thus, the specific surface area) within the lattice. For lattice cylinder electrodes with 55, 89, and 111 cm^2^ geometric surface area, simple cubic lattices with specific surface area 0.10, 0.12, and 0.15 cm^2^/cm^3^ were used, respectively. Electrodes were designed to occupy a cylinder with dimensions 4 cm height by 2.4 cm diameter. Molds based on the electrodes were created by inverting the design; the molds were printed at 135% of the designed size, to account for shrinkage of the material during carbonization of the organic aerogel to form the carbon aerogel.

Carbon aerogels were prepared from resorcinol and formaldehyde (RF) gels. In a typical synthesis, resorcinol (1.23 g) and a 37% formaldehyde solution (1.7 g) in water (1.5 ml) were mixed together, followed by addition of 2N nitric acid (44 μl). The mixture was poured into a custom 3D-printed ABS-mold, which was placed in a sealed glass jar and allowed to cure in an oven at 80°C for 24 h to form wet organic aerogels. After curing, the ABS mold was completely dissolved in a bath of acetone. The acetone bath also served to solvent exchange the organic aerogels. Typically, the acetone was refreshed 2 or 3 times to fully remove the ABS mold. The organic aerogels were then allowed to dry in the fume hood for 24 h. The dried aerogels were carbonized in a tube furnace under nitrogen atmosphere at 1,050°C for 3 h with a heating and cooling rate of 2°C/min to obtain carbon aerogels. The CA electrodes were attached to a copper wire as a current collector using conductive Ag epoxy, which was then sealed with a non-conductive epoxy. The copper wire was insulated to ensure the electrochemical activity would occur only at the electrode.

The CA electrodes were electroplated with a catalyst-layer of nickel-molybdenum for enhanced hydrogen evolution properties under biological conditions. For this, each cathode was placed in NiMo-plating solution at ∼1 cm distance to a surrounding anode (Platinum mesh, PT008720 -350-210-23, Goodfellow), which was separated from the cathode using nylon filter bags (180 micron mesh, SLSON, Amazon.com). NiMo-plating solution contained per liter: 40 g NiCl_2_⋅6H_2_O, 25 g Na_2_MoO_4_⋅2H_2_O, 45 g sodium citrate. The pH was adjusted to 10 with NH_4_OH. Under constant magnetic stirring at 900 rpm, a constant current of 50 mA/cm^2^ was applied to obtain a deposition of 60 Coulombs/cm^2^. The cathodes were soaked in deionized water to remove residues from the NiMo-plating before use in the bio-electrochemical system.

### Microbial Strain, Growth Medium, and Culture Conditions

The archaeon *M. maripaludis* was chosen due to its salinity tolerance and its exceptional performance in previous studies (Kracke et al., 2020). *M. maripaludis* was cultured using chemically defined medium-JD specifically modified for use in bio-electrochemical reactors as reported previously (Kracke et al., 2020). In brief, medium-JD contained per liter: 30 g Na_2_SO_4_, 4 g MgSO_4_ × 7 H_2_O, 0.2 g KH_2_PO_4_, 0.4 g NH_4_HCO_3_, 0.6 g KHCO_3_, 0.04 g CaCl_2_, 7.2 g morpholinepropanesulfonic (MOPS) acid, 3.4 g MOPS Na-salt, 1 ml selenite-tungstate solution, 1 ml trace element solution SL-10, 1 g NaS_2_O_3_, 0.4 g Cysteine-HCl and 30 mL of 1 M NaHCO_3_ solution. The final medium-JD was prepared from autoclaved anoxic stock solutions under continuous gassing with CO_2_/N_2_ (20/80% v/v). The final pH was 6.8.

*Methanococcus maripaludis* was routinely grown in batch cultures in medium-JD under H_2_/CO_2_ (80/20 %v/v) gas atmosphere at 30°C and 250 rpm. Each reactor was inoculated from an exponentially growing culture.

### Reactor Set up and Operation

The bioelectrochemical set up was described in detail previously (Kracke et al., 2019). In brief, two-chamber glass H-cells were separated by a Nafion 117 proton-exchange membrane (Fuel Cell Store Inc., College Station, TX, United States, surface area 4.9 cm^2^). The volume of cathode and anode chamber was 100 mL, respectively. In each reactor, one three-dimensional cathode, fabricated as reported above, was inserted into the cathode compartment and connected by threading the copper wire through a gastight rubber stopper. The anode was a platinized titanium mesh (1′′ × 4′′, TWL, Amazon) and the reference electrode Ag/AgCl (NaCl saturated; BASI). The electrochemical reactors were controlled by applying a constant current using a multichannel potentiostat (VMP3; Bio-Logic Science Instruments, France). For experiment 1 and 2 the current was set to 50 mA per reactor and for experiment 3 the current was increased stepwise in increments of 10 to 60 mA, 70 mA and finally 80 mA. Each time, the system was monitored until *steady state* conditions were reached, which was defined at a minimum of three consecutive measurements of constant methane production rate (*cf.* Figure 4). The pH in the catholyte was monitored and remained constant (pH7) at all times.

CO_2_ was supplied continuously at a flowrate of 0.15 mL min^–1^ (CO_2_ 100%) controlled via mass flow controllers (EL-Flow F-100D, Bronkhorst) to ensure non CO_2_-limiting conditions throughout the experiments. The microbial growth medium-JD was continuously supplied via peristaltic pump at a constant feed rate of 0.0676 ± 0.0025 mL min^–1^. Each chamber was magnetically stirred at 700 rpm. All reactors were operated at ambient pressure in a temperature-controlled room at 30°C. Prior to inoculation the system was operated for 72 h to reach stable pH conditions in the cathode chambers [see detailed description elsewhere (Kracke et al., 2020)].

Liquid samples were taken through rubber side ports at regular intervals. Samples for gas analysis were taken from the respective reactor headspace with a gas syringe. Volumetric gas flow rates of the reactor off gas were measured via Milligas counter (KG MGC-1, Ritter Apparatebau GmbH & Co., Germany) at regular intervals.

### Analytical Methods

Methane and hydrogen were measured using a gas chromatograph (equipped with a thermal conductivity detector and a flame ionization detection detector, Agilent 6890N, Agilent, Santa Clara, CA, United States). Gaseous headspace samples were taken in regular intervals from the cathode chamber with a gastight syringe (VICI). For each sample, 300 μl were injected into a gas chromatograph equipped with a thermal conductivity detector and a flame ionization detection detector. Analysis of methane and was made via the flame ionization detector. Separation of CH_4_ and H_2_ was accomplished in a GS-Q capillary column (30 m length, 0.530 μM ID) and helium was used as carrier gas at a flow rate of 7.4 ml min^–1^. The injector (split ratio 0.1:1) and flame ionization detector temperatures were 250°C, and analyses were isothermal at 100°C column temperature.

Cell densities were measured via Spectroscopy at 600 nm (Ultrospec 2100 pro, Amersham BioSciences, Little Chalfont, United Kingdom).

### Calculations

Coulombic efficiencies were calculated by dividing the electrons recovered in methane by the electrons supplied as current at a certain time point according to equation 1 below. With η_(__*CH*__4,t__)_ = mol CH_4_ at time point t; *f*_(__*e*_,_*CH4*__)_ = molar conversion factor (8 electrons per mol CH_4_); *F* = Faraday constant (96,485 C mol^–1^ of electron) and I = electric current.

(1) $$CE\left[\%\right]=\frac{\eta_{C\,H\,4,t}\times f_{e,C\,H\,4}\times F}{\int_{t\,0}^{t}I\,dt}\times 100$$

The estimation of the current density at which bubble formation occurs was done using Fick’s law (Eq. 2) and the following assumptions. The diffusion coefficient (D) for hydrogen in water is 5.5^∗^10^–5^ cm^2^ s^–1^ (atmospheric pressure, 30°C) (Verhallen et al., 1984). The solubility limit for hydrogen at 30°C in water is 0.75 mM (Verhallen et al., 1984). Assuming complete utilization of H_2_ by hydrogenotrophic microbes in the bulk liquid (∂⁡φ = 0.75 mM) and a diffusive boundary layer of thickness ∂⁡*x* = 100 μm (Kracke et al., 2019).

$$J=-D\,\frac{\partial \varphi }{\partial x}=-5.5\cdot 10^{-5}\,\frac{0.75}{0.01}\,\left[\frac{c\,m^{2}}{s}\cdot \frac{m\,m\,o\,l}{1000\,c\,m^{3}\,c\,m}\right]$$

(2) $$=-4.125\cdot 10^{-6}\,\left[\frac{m\,m\,o\,l}{s\,c\,m^{2}}\right]$$

Therefore, in good approximation 4.1 nmol s^–1^ H_2_ will diffuse away from the electrode per cm^2^. This corresponds to a maximum current density of around 0.8 mA/cm^2^ before bubble formation occurs in this system.

## Results

### 3D-Printing for Fabrication of Electrode Prototypes for *in situ* H_2_ Delivery at Variable Current Densities

To test how the cathodic current density influences process parameters in hydrogen-driven microbial electrosynthesis, we used additive manufacturing to design and fabricate hydrogen-evolving cathode prototypes with identical catalytic properties and overall volume but varying surface areas.

The base material was comprised of carbon aerogel, which exhibits a high electrical conductivity, and good structural stability (Chandrasekaran et al., 2018). After the final synthesis step of carbonization at high temperature in inert atmosphere, the cathodes were electroplated with NiMo-alloy to enhance the hydrogen evolution properties, as described previously (see Figure 1A) (Kracke et al., 2019). Sets of electrodes that share identical overall cylindrical volume of 18 cm^3^ were fabricated with varying geometric surface areas 55, 86, and 111 cm^2^ by controlling the density of lattice struts contained within the cylinder (Figures 1B,C). A filled cylinder cathode with the geometric surface area of 39 cm^2^ was prepared via NiMo-plating of a commercial graphite rod with the same overall cylinder dimensions, as the bulk cylinder CA fabrication did not result in uniform shapes due to uneven shrinkage and cracking during drying and carbonization.

### Electromethanogenesis From *in situ* Produced H_2_ Supplied at Varying Current Densities

The novel 3D cathodes were employed in an integrated bioelectrochemical reactor as described previously (Kracke et al., 2020). In brief, four identical H-cell reactors were operated in *chemostat-mode* under continuous supply of gaseous CO_2_ and fresh, sterile growth medium. The supply of catabolic electrons was controlled by applying a constant current of 50 mA per reactor. Cathodes evolved H_2_ at 100% selectivity (Kracke et al., 2019) and, therefore, supplied equal amounts of H_2_ to each reactor. The current density varied based on the available cathode geometric surface area; the tested specific current densities were 0.5, 0.6, 0.9, and 1.3 mA/cm^2^. After an abiotic equilibration period of 72 h, each reactor was inoculated with the anaerobic archaeon *M. maripaludis* to an initial optical density of OD_600, start_ = 0.02.

**FIGURE 2:**
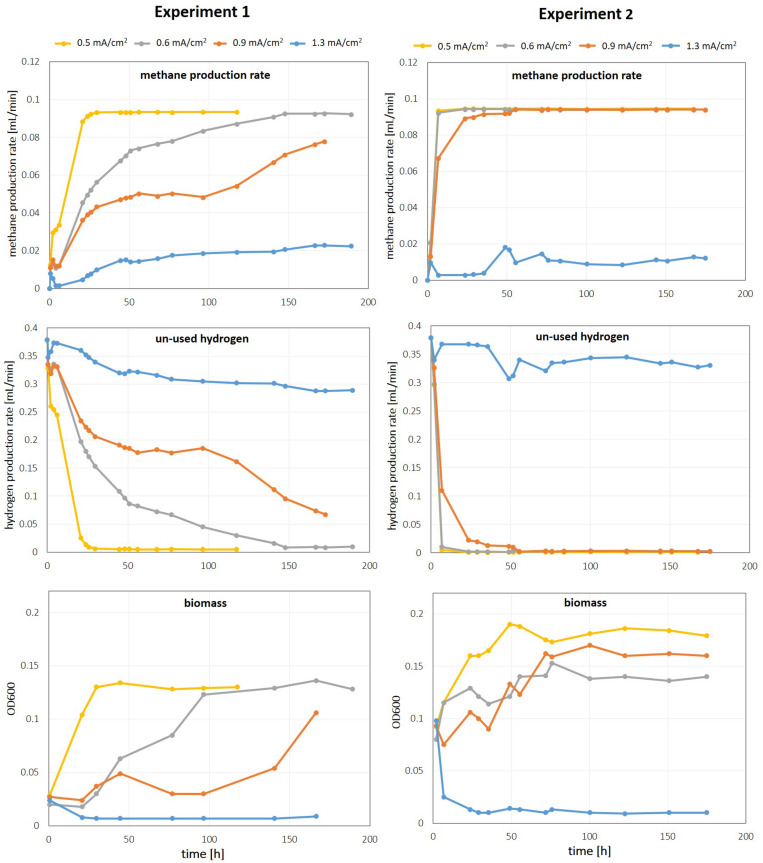
Methane production rate, unused hydrogen in the reactor off gas and microbial growth during Exp1 (initial OD = 0.02, left column) and Exp2 (initial OD = 0.1, right column).

The methane production profiles of the different reactors during this first experiment indicated that efficient electromethanogenesis in this system strongly depended on the current density of the cathode (see Figure 2, Exp1, left column). At the lowest current density of 0.5 mA/cm^2^ (high surface area), stable electromethanogenesis was observed within 24 h reaching an average coulombic efficiency of 98 ± 0.2%. With increasing current density, *steady state* operation was delayed and H_2_ concentration in the reactor off-gas increased. At 0.6 mA/cm^2^ highly efficient methane production was established only after 145 h (CE = 97 ± 0.4%), while the reactor operating at 0.9 mA/cm^2^ did not reach stable methane production rate within the monitored timeframe of 1 week. At the highest current density of 1.3 mA/cm^2^ tested, substantial underutilization of H_2_ was observed, which resulted in an overall low coulombic efficiency of 20 ± 4% for methane (averaged between 50 and 190 h). These data demonstrate that the cathodes fabricated with 3D-printing technology are suitable for direct integration with microbial electrosynthesis; however, the overall start-up time and coulombic efficiency strongly depended on the cathodic current density.

### Effect of Initial Cell Concentration on Reactor Performance

**FIGURE 3:**
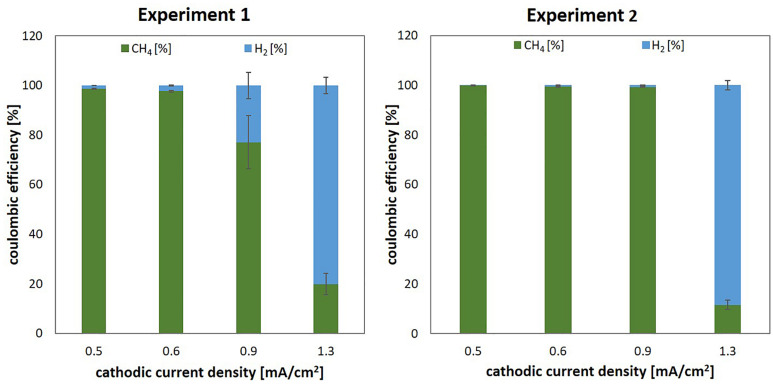
Coulombic efficiencies achieved with the 3D printed cathodes of different surface areas under low cell density (Exp1, left) and high cell density conditions (Exp2, right). The given values are averages of 4–12 individual measurements taken after reaching *steady state*, with error bars displaying the standard deviation between individual measurements.

To investigate whether the observed effect of current density on start-up times during experiment 1 was dependent on the initial cell density, we repeated the experiment (identical set-up, unused set of cathodes) and increased the initial cell density by a factor of 5 to an OD_600, start_ = 0.1 (Exp2). At a higher initial cell density, start-up times were significantly shortened but lower current densities still resulted in improved overall process performance (see Figure 2, Exp2, right column). For current densities of 0.5 and 0.6 mA/cm^2^, highly efficient methane production was achieved within less than 7 h (for coulombic efficiencies see Figure 3). At a current density of 0.9 mA/cm^2^, *steady state* methane production was reached after 55 h, while the low-inoculum culture of Exp1 did not reach stable production rates within 7 days at this current density. Apparently, electromethanogenesis at the highest current density of 1.3 mA/cm^2^ tested did not significantly benefit from a high initial cell density. Again, significant amounts of H_2_ were lost in the reactor off gas and the average coulombic efficiency for methane was only 12 ± 2% (cf. Figure 3).

While increasing cell density significantly shortened start-up times, the achieved coulombic efficiency in *steady state* conditions was dependent on the cathodic current density.

### Current Density as a Critical Determinant for Biocompatibility

**FIGURE 4:**
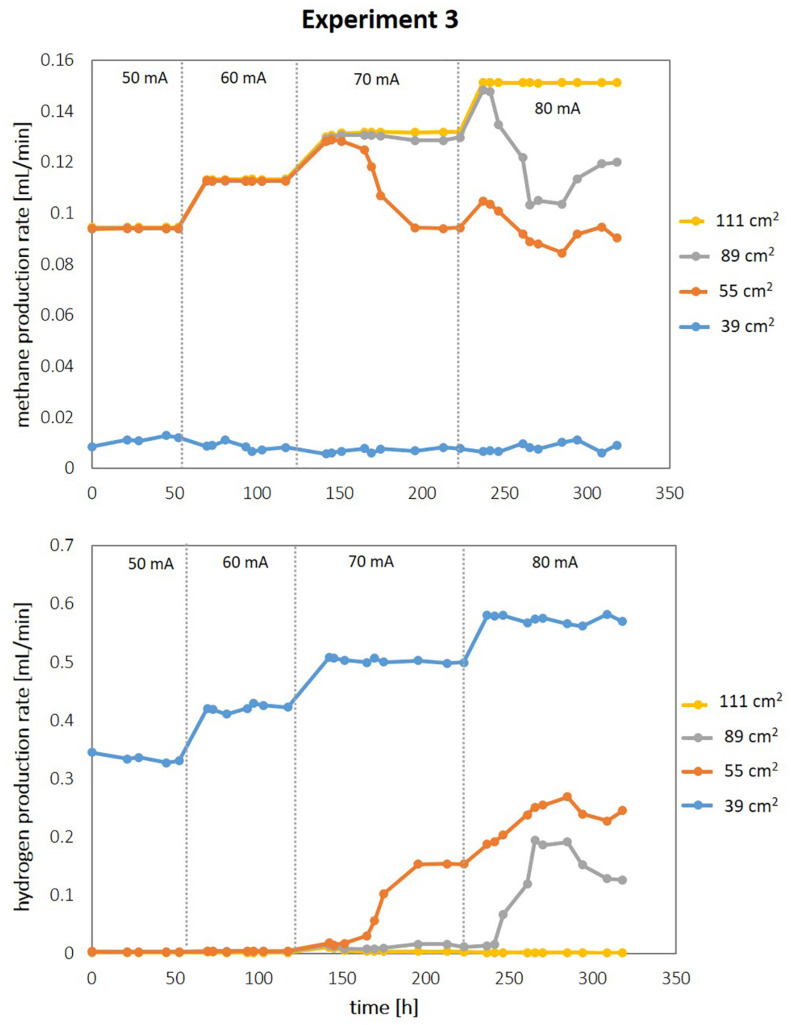
Effect of increasing current density on electromethanogenesis performance in reactors with different cathodic surface areas. Methane production rate and unused hydrogen in the reactor off gas during Exp3. The total supplied current was increased stepwise as indicated per dashed vertical lines. Initial OD = 0.1.

**FIGURE 5:**
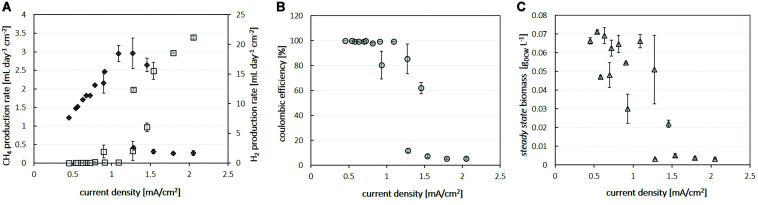
Summary of process parameters during electromethanogenesis in dependency of the applied cathodic current density: **(A)** The rates of methane (diamonds, left axis) and H_2_ (squares, right axis) produced per cathode area, **(B)** coulombic efficiencies for methane (circles) and **(C)** *steady state* biomass concentrations (triangles). Eight individual electrodes of four different surface areas 39, 55, 86, and 111 cm^2^ were tested under constant current conditions of 50, 60, 70, and 80 mA. The given values are averages of 4–12 individual measurements under *steady state* conditions with error bars displaying the standard deviation between individual measurements.

Based on the above experiments, we hypothesized that the specific current density on the cathodes is crucial for efficiently delivering H_2_ *in situ* into the microbial metabolism. To test this hypothesis, we increased the total current per reactor and, therefore, the current density in three consecutive steps in the following experiment 3 (Figure 4). With increasing current densities, the efficiency of bioelectrochemical methane production decreased as the H_2_ concentration in the off-gas increased. At 60 mA total current, the reactors with the three highest surface area cathodes enable methane production at almost 100% coulombic efficiency. Increasing the current to 70 mA led to an increase in methane production rate, except in the reactor with the second smallest surface area of 55 cm^2^, where the methane production rate dropped slightly instead (*cf.*, Figure 4). A further increase in total current to 80 mA resulted in a similar effect for the next larger cathode at 89 cm^2^. Under these conditions only the cathode with the largest surface area (111 cm^2^) resulted in a further increase in methane production rate, corresponding to a volumetric methane production rate of 2.2 L_*CH4*_ /L_*catholyte*_/day at a coulombic efficiency of 99%. These observations suggest that after exceeding a critical current density the performance ceases to increase further and, more significantly, drops as highly efficient electromethanogenesis is no longer observed (see Figure 5). For the here tested system using NiMo cathodes, magnetic stirring (700 rpm) and *M. maripaludis* as microbial catalyst and at the cell densities tested, this critical current density was found to be around ∼1.0–1.5 mA/cm^2^ (*cf*. Figure 5). At lower current densities, methane production rates per electrode area increase linearly with increasing current densities (Figure 5A), which indicates highly efficient conversion of the supplied electrical current into methane under these conditions resulting in near 100% coulombic efficiencies (Figure 5B). When current densities between 1.0 and 1.5 mA/cm^2^ were applied, the product spectrum shifted from methane to hydrogen, which resulted in a drop of coulombic efficiency (Figures 5A,B). At current densities exceeding 1.5 mA/cm^2^, hydrogen was the major product, increasing linearly per area with the applied current density (Figure 5A). Under these conditions, the cell concentration in the reactor dropped significantly (Figure 5C), despite the increasing amount of hydrogen being produced. These data demonstrate that the efficient *in situ* delivery of H_2_ in the bio-electrochemical system strongly depends on the cathodic current density. Large surface area cathodes that enabled current densities to remain below the observed critical current density threshold were successful to facilitate continuous electromethanogenesis via *in situ* H_2_ production at record volumetric production rate. The underlying mechanism for this observed behavior is likely a combination of increased loss of gaseous H_2_ due to increased bubble formation and decreased biocompatibility due to locally high pH at the cathode surface at higher current densities and is discussed in detail in the following section.

## Discussion

### Mechanism of Efficient H_2_ Delivery in MES

**FIGURE 6:**
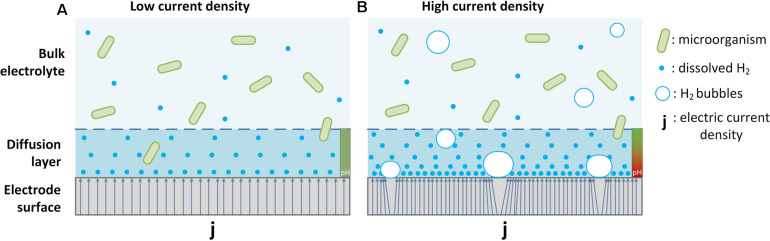
Conceptual representation of the fate of H_2_ in a **(A)** single-phase system at low current density and **(B)** gas-evolving system at high current density on a hydrogen producing cathode during electrosynthesis. The concentration of dissolved H_2_ in the bulk aqueous phase is lower than in the boundary layer because of microbial consumption. If the cathodic current density produces dissolved H_2_ at a rate that exceeds its diffusion out of the boundary layer and microbial consumption, bubble formation will occur **(B)**. Hydrogen gas bubbles can passivate the active catalytic surface area of the cathode and, thus, reduce electric efficiencies. If mass transfer from gaseous H_2_ in bubbles to dissolved H_2_ in the bulk electrolyte is limited, there is a net loss of H_2_ from the reaction space which reduces coulombic efficiency as well as volumetric production rates. When the electrocatalytic H_2_ production exceeds the rate at which protons diffuse into the boundary layer, the local pH will rise to alkaline levels (indicated red, see **B**) potentially toxic to the microorganisms. With increasing current density, number and size of bubbles will increase and with that the intensity of all associated effects.

Similar observations of optimum performance in MES at relatively low current densities have been reported for other systems (Bajracharya et al., 2015; Blanchet et al., 2015). In an electromethanogenesis system, Geppert et al. (2019) increased the current density from 0.5 to 3.5 mA/cm^2^ and found optimum conditions at around 2.5 mA/cm^2^, while at higher current densities the fraction of electrons lost as unused hydrogen in the off-gas increased. This was interpreted by others as a “metabolic limitation of the biocatalyst” (Dessì et al., 2020). However, microbial production of methane as well as acetate from H_2_/CO_2_ is possible at rates exceeding the rates achieved in bioelectrochemical systems today by about one order of magnitude (Rittmann et al., 2012; Kantzow et al., 2015; Asimakopoulos et al., 2018). This demonstrates that the metabolic capacity of the microorganism is not limiting. Instead, our data show that the mode of H_2_ delivery ultimately determines the fraction of electrons available for microbial metabolism, which, therefore, determines the maximum achievable production rate under the given conditions. For gas fermentation, it is well known that efficient delivery of dissolved H_2_ is a key limiting factor for maximizing production rates, and, therefore in some cases, requires expensive pressurization to increase H_2_ dissolution (Phillips et al., 2011, 2017; Asimakopoulos et al., 2018). Thus, the electrochemical production of H_2_ *in situ* can provide substantial advantage over introducing and dispersing bulk H_2_ gas if engineered well (*cf*. Figure 6).

Hydrogen-driven electrosynthesis is mainly performed by cells in suspension in the reactor bulk liquid, where turbulent mixing enhances mass transfer of cathode-derived H_2_ while p_(__*H2*__)_ is effectively drawn close to zero by microbial metabolism (Conrad and Wetter, 1990; Kotsyurbenko et al., 2001; Feldewert et al., 2020). The electrode surface presents a liquid-solid interface, around which a diffusive boundary layer exists. In a magnetically stirred (200 rpm) electromethanogenesis reactor, we previously determined the boundary layer around a graphite rod cathode to be ∼50–100 μm (Kracke et al., 2019). Therefore, electrocatalytically produced H_2_ needs to pass through this diffusion-limited boundary layer before it is available to the microbial culture in bulk liquid (see Figure 6). At low current density, the rate of formation of dissolved H_2_ is slower than microbial consumption in and diffusion out of the boundary layer, and therefore no H_2_ gas bubbles form (Figure 6A). When the current density increases and the rate of hydrogen formation exceeds H_2_ consumption and diffusion out of the boundary layer, the solubility limit of H_2_ is reached and gas bubbles start to form (Figure 6B). Such bubble formation in electrosynthesis processes is generally undesirable as they can (i) passivate the active sites of the electrocatalyst reducing the electric efficiency and (ii) escape the reactor unused and, therefore, reduce coulombic efficiencies and lower the purity of output gas products (Figure 6B) (Angulo et al., 2020). While H_2_ present in bubbles can still be available for metabolic conversion in the reactor’s bulk liquid depending on the geometry and operation, as previously noted for gas fermentation, the size and number of bubbles increases with current density. A video documentation of this process demonstrates the difference in hydrogen bubble formation under constant current conditions on the here tested cathodes with varying surface areas (see Supplementary Information to this article). It can be seen that at identical overall H_2_ production rate (identical current), the cathodes with lower surface areas lead to significantly increased bubble formation compared to higher surface area cathodes (*cf.* Supplementary Video 1–3). Based on our calculation using Fick’s law and the constraints of our system (see section “Materials and Methods” for details), formation of H_2_ bubbles is predicted to start at currents densities exceeding 0.8 mA/cm^2^. This can explain the observation of significant H_2_ loss at current densities above 1.0 mA/cm^2^ in the here tested system (*cf.* Figure 5).

A second, often underexplored aspect of the boundary layer in bioelectrochemical systems is the formation of a pH gradient across the boundary layer and its effect on microbial metabolism. In circumneutral microbial growth medium the concentration of H^+^ and OH^–^ ions is low at 0.1 μM (pH = 7). Therefore, electrochemical proton reduction to H_2_ at even moderate current density can lead to high local pH in the diffusive boundary layer (Jayathilake et al., 2018). While the presence of a buffer in mM concentration can significantly mitigate this effect (Auinger et al., 2011; Shinagawa et al., 2019), the rate of diffusion of the protonated buffer into the boundary layer from the bulk liquid becomes limiting for a sustained formation of dissolved, bubble-free H_2_ at medium or high current densities (Figure 6B). An alkaline pH in the boundary layer in combination with bubble formation presumably precludes the formation of biofilms at high current densities (Blanchet et al., 2015). Although the largest fraction of microbial activity during H_2_-driven MES is in the bulk liquid, metabolically active microbes do come in contact with the high pH environment in the cathodic boundary layer. Exposure to alkaline pH environments can compromise microbial organisms like *M. maripaludis* (Schauer and Whitman, 1989). In fact, in a previous study we found slightly increased cell lysis of *M. maripaludis* cells grown with *in situ* electrochemically evolved hydrogen (at 1 mA/cm^2^) compared to cultures supplied with a gaseous mix of H_2_/CO_2_ (Kracke et al., 2020).

In combination, the increased H_2_ loss through bubble formation and decreased biocompatibility due to high local pH can explain the here observed decline in biomass and methane formation rates after exceeding a critical current density. The drop in methane formation rate seems to slightly precede the decrease in biomass concentration (*cf.* Figure 5), indicating that in the here investigated system, H_2_-loss via bubble formation is the dominating effect before bio-incompatible pH conditions are reached at further increased current densities. The specific value of this current density threshold depends for each MES system on its specific properties, including reactor configurations, operating conditions, boundary layer and mixing, and microorganisms chosen. To maximize the microbial production rate per catalytic electrode area, it would be beneficial to operate a microbial electrosynthesis reactor close to its critical current density (*cf.* Figure 5A). Thus, for designing advanced MES systems, it is of critical importance to consider the above discussed molecular basis of bubble formation and local pH gradients. Because these gradient effects increase with the thickness of the diffusive boundary layer (Vogt, 1980), reducing the size of this layer will mitigate these limitations and maximize the supply of electrons per reactor volume while maintaining biocompatibility. 3D-printing is a particularly promising platform to effectively decrease the boundary layer via advanced electrode and reactor designs that combine high surface areas with improved fluid dynamic properties (Lee et al., 2019) and, therefore, presents a critical opportunity to further improve the performance of MES.

## Conclusion

We demonstrated here for the first time electrocatalytically active cathodes fabricated via 3D-printing as a new platform for efficient *in situ* delivery of H_2_ in an integrated microbial electrosynthesis system. With this tool, we identified the specific current density on the cathode surface as being critical for (i) fast start-up times of the microbial culture, (ii) stable steady-state performance, and (iii) high coulombic efficiencies via minimizing H_2_ escape, favoring efficient microbial H_2_ utilization, and maintaining biocompatible pH conditions near the electrode surface. Moreover, we showed that it is not the metabolic capacity of a microorganism *per se* that limits product formation rate in a MES, but the physical/chemical conditions in the boundary layer.

This proof-of concept demonstrates that advanced manufacturing of cathodes is a suitable tool for bioelectrochemical technologies, which provides major advances for the rapid development of electrode-prototypes and enables advanced custom design of innovative bio-electrochemical reactors. Even in our non-optimized H-cell reactor the cathodes with highest surface area enabled highly efficient electromethanogenesis at record volumetric rate of 2.2 L_*CH4*_ /L_*catholyte*_/day (111 cm^2^, – 80 mA, CE = 99%). Future research efforts will show how this new platform can be used to develop microbial electrosynthesis systems via *in situ* H_2_ production that provide superior mass transfer conditions compared to a two-step process of water electrolysis and subsequent gas fermentation.

## Data Availability Statement

The original contributions presented in the study are included in the article/Supplementary Material, further inquiries can be directed to the corresponding authors.

## Author Contributions

FK designed and performed the experiments in the integrated bio-electrochemical system, analyzed the data, and drafted the manuscript. BJ and SC fabricated the electrodes. SP designed the electrodes and molds. FK, JD, BJ, SP, SC, SB, and AS conceived and designed the study and contributed in regular data analyzes and discussions. All authors edited, read, and approved the final manuscript.

## Conflict of Interest

The authors declare that the research was conducted in the absence of any commercial or financial relationships that could be construed as a potential conflict of interest.

## Publisher’s Note

All claims expressed in this article are solely those of the authors and do not necessarily represent those of their affiliated organizations, or those of the publisher, the editors and the reviewers. Any product that may be evaluated in this article, or claim that may be made by its manufacturer, is not guaranteed or endorsed by the publisher.

## Supplementary Material

Supplementary Video 1 39 cm^2^ at –50 mA.

Supplementary Video 2 55 cm^2^ at –50 mA.

Supplementary Video 3 111 cm^2^ at –50 mA.
